# Long-term implant retention after impacted elastic stable intramedullary nailing in pediatric diaphyseal forearm fractures: a retrospective cohort study

**DOI:** 10.2340/17453674.2026.45693

**Published:** 2026-04-01

**Authors:** Jan Egil BRATTGJERD, Christer AASHEIM, Astrid ROSENBERG, Christoffer FOTLAND, Vera HALVORSEN

**Affiliations:** 1 Division of Orthopaedic Surgery, Oslo University Hospital, Oslo; 2 Institute of Clinical Medicine, Faculty of Medicine, University of Oslo, Oslo; 3 Institute of Rehabilitation Science and Health Technology, Faculty of Health Science, Oslo Metropolitan University, Oslo, Norway

## Abstract

**Background and purpose:**

In elastic stable intramedullary nailing (ESIN) for forearm fractures, nail protrusion may cause irritation-related implant removal. The primary aim was to evaluate implant retention after nail impaction, and secondarily to assess secondary procedures, complications, and protrusion as a predictor of removal.

**Methods:**

We analyzed a retrospective cohort of children with diaphyseal forearm fractures treated with impacted ESIN between 2017 and 2024. Implant retention was defined as no nail removal at record review. Secondary procedures were unplanned operations, while complications were adverse events managed conservatively. Radiographic nail protrusion was measured as extraosseous nail length and evaluated using receiver operating characteristic analysis to predict irritation-related implant removal.

**Results:**

160 children with diaphyseal forearm fractures were included. At a mean observation time of 5 years, 132/160 children retained their implants (83%, 95% confidence interval [CI] 76–88). Secondary procedures occurred in 30/160 patients (19%, CI 13–26), most commonly irritation-related implant removal (10%, CI 6–16) and refracture (6%, CI 3–10). Complications occurred in 19/160 patients (12%, CI 7–18), including superficial radial nerve symptoms (7/160; 4.4%) and irritation without implant removal (6/160; 3.8%). A protrusion threshold of 3.3 mm predicted irritation-related implant removal (AUC 0.79, CI 0.71–0.86; sensitivity 100%, CI 83–100; specificity 55%, CI 49–61). No irritation-related removals occurred below this threshold.

**Conclusion:**

Impacted ESIN was associated with high long-term implant retention, although secondary procedures and complications occurred in about one-third of the patients. A protrusion threshold of 3 mm was linked to irritation-related removal and may guide implant retention.

In children, elastic stable intramedullary nailing (ESIN) is a standard method for stabilizing long-bone fractures [1], and is most frequently used in the forearm [2]. The nails preserve the physes and provide flexible 3-point fixation [3], while their ends are traditionally left slightly protruding to facilitate later implant removal. This practice provokes local irritation at the ESIN entry site, the most common complication [4], reported in up to 19% before implant removal [5].

The rationale for routine removal is largely historical, rooted in the original recommendations of the Nancy group [6], the tradition of implant removal in children once bone healing is complete [7], and concerns regarding corrosion, stress shielding, difficult extraction, and adverse tissue reactions [8]. While routine removal is generally safe, complications such as failed extraction and approach-related superficial radial nerve symptoms have been reported [9,10]. It is not recommended before 6 months, as earlier removal increases refracture risk [3]. Although most patients are asymptomatic at this time point [9], routine removal remains common [1] but has been questioned [11]. Given the burden of secondary procedures that may be unnecessary for most patients, implant retention has been proposed for asymptomatic patients [12]. However, evaluation in the forearm has so far been limited to small series [13].

Technical refinements, with nail trimming and axial advancement using an impactor, have been proposed to reduce irritation and limit the need for removal [11]. Nevertheless, long-term data on implant retention after such techniques remain sparse, and it is unclear to what extent residual nail protrusion is compatible with implant retention without local discomfort. Consequently, guidance supporting retention rather than routine removal is limited.

We evaluated implant retention, secondary procedures, and complications after impacted ESIN in pediatric forearm fractures, and assessed the discriminative ability of postoperative nail protrusion to predict irritation-related implant removal.

## Methods

### Study design

We conducted a single-center retrospective cohort study at Oslo University Hospital, a tertiary referral center. Given the observational, population-based design, no power calculation was performed. Consecutive inclusion and a standardized data extraction protocol were used to reduce selection and information bias, respectively. Reporting followed the STROBE guidelines [14].

### Participants

Eligible patients were identified through the institutional patient administrative system using procedure codes for intramedullary fixation of diaphyseal forearm fractures. Between January 1, 2017, and December 31, 2024, children younger than 16 years were included, provided that follow-up occurred within the hospital catchment area.

### Follow-up protocol

Patients were routinely followed until clinical and radiographic union. Postoperative follow-up adhered to a standard schedule at 2 weeks (cast removal and radiographic alignment), 6 weeks (radiographic union and range of motion), and 12 weeks (functional outcome), or later when indicated. Follow-up was considered complete when no further visits were required. Follow-up time was defined as the interval from the index operation to the last appointment, while observation time was defined as the interval from the index operation to the date of review. For patients without further contact, a later uneventful course was assumed, as all forearm fracture surgery within the catchment area is performed at this institution. Postoperative care was supervised by a pediatric orthopedic surgeon.

### Data collection

Data was extracted from medical records using a predefined protocol based on previous studies [1,3]. Collected variables included demographics, fracture characteristics, treatment details, secondary procedures, complications, and duration of follow-up. Fracture variables comprised side, diaphyseal single- or both-bone fracture pattern, and open vs closed injury. Indications for operative treatment were displaced diaphyseal forearm fractures, treated either primarily or secondarily, when acceptable alignment could not be maintained after closed reduction and casting. Treatment variables included reduction method, approach, number and diameter of nails, and operative time.

### Outcomes

The primary outcome was implant retention, defined as the absence of nail removal at record review. Secondary outcomes were secondary procedures and complications, as identified in the medical records. Secondary procedures were defined as unplanned operations, and complications as treatment-related adverse events managed without surgery. Nail protrusion was evaluated as a predictor of implant removal due to local nail-end irritation. Irritation at the ESIN entry site was defined as local symptoms (pain and/or activity-related discomfort) and/or clinical signs (tenderness on palpation).

### Surgical technique

Operations were performed under general anesthesia with the patient supine and the arm on an arm table. Open reduction was reserved for cases with unacceptable alignment after closed reduction. Titanium elastic nails (TEN; DePuy Synthes, Oberdorf, Switzerland) were pre-bent and inserted through small metaphyseal incisions approximately 1–2 cm from the growth plate, retrograde in the radius and antegrade in the ulna. Nails were cut in situ and subsequently impacted by axial advancement using an impactor to reduce extraosseous length and clinical prominence. No predefined target for nail protrusion was used, and seating was based on intraoperative assessment. Alignment and nail position were verified fluoroscopically. An above-elbow cast was applied for analgesia. After cast removal, active motion with restricted loading was encouraged until bone healing, with instructions provided at outpatient visits. All procedures were performed by orthopedic residents with at least 3 years of training. This retention strategy was introduced as a technical refinement without formal training and has been used at our institution since 2015.

Secondary procedures and complications were further categorized as early occurring (< 1 year) or late (> 1 year). Soft-tissue complications included local irritation prompting reassessment or implant removal. Postoperative nerve deficits were considered treatment related. Bone-healing disturbances included delayed union (3–6 months without bridging callus), non-union (> 6 months), refracture (including re-displacement before bone consolidation), malalignment (> 10° on radiographs), and loss of forearm rotation (> 20°). Infections were classified according to the need for surgical intervention.

**Figure 1:**
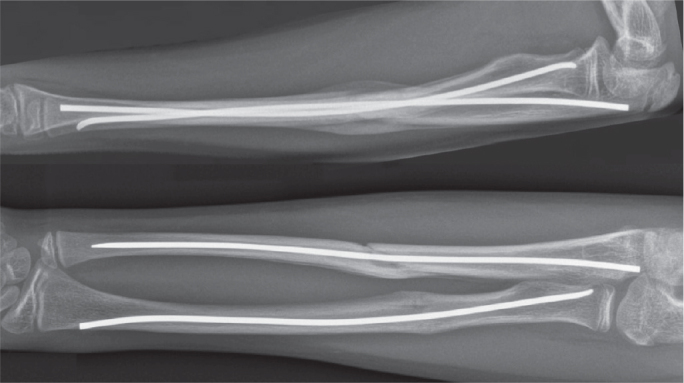
Postoperative lateral and anteroposterior radiographs of the forearm 6 weeks after elastic stable intramedullary nailing, showing fracture alignment with callus formation and adequate axial nail impaction in both the radius and ulna, with no extraosseous nail protrusion.

Nail protrusion was defined as the extraosseous nail length, measured from the cortical surface to the outer nail end, and calibrated to the known nail diameter (Figure 1) [5,11,15]. Measurements were performed by an orthopedic resident on postoperative radiographs obtained within 1 week, following group consensus. Radial and ulnar measurements were recorded separately but analyzed together.

### Statistics

Statistical analyses were performed using SPSS version 30 (IBM Corp, Armonk, NY, USA). The primary and secondary endpoints were analyzed descriptively. In addition, nail protrusion was evaluated as a predictor of irritation-related implant removal. Categorical outcomes were summarized as proportions with 95% confidence intervals (CIs) and compared using Fisher’s exact test, with odds ratios and CIs. Continuous variables were presented as medians with interquartile ranges (IQRs) and compared using the Mann–Whitney U test. Receiver operating characteristic (ROC) analysis was performed at the nail level to evaluate the discriminative ability of postoperative nail protrusion to predict irritation-related implant removal. Nails removed for indications other than irritation were excluded from this analysis to avoid misclassification. Protrusion was treated as a continuous predictor, and discrimination was quantified using the area under the curve (AUC) with CIs. The optimal threshold was identified using Youden’s index, and sensitivity and specificity with CIs were calculated at this threshold in the same dataset. Absolute cell counts underlying these estimates were obtained.

### Ethics, funding, use of AI tools, and disclosures

The study was approved by the Regional Committee for Medical and Health Research Ethics (approval no. 2025/874311) and conducted in accordance with applicable standards. ChatGPT was used during manuscript revision for language polishing. The tool was not used to generate scientific content, analyses, or interpretations. The authors take full responsibility for the final manuscript. No external funding was received, and the authors declare no conflicts of interest. Complete disclosure of interest forms according to ICMJE are available on the article page, doi: 10.2340/17453674.2026.45693

## Results

### Study population

**Figure 2:**
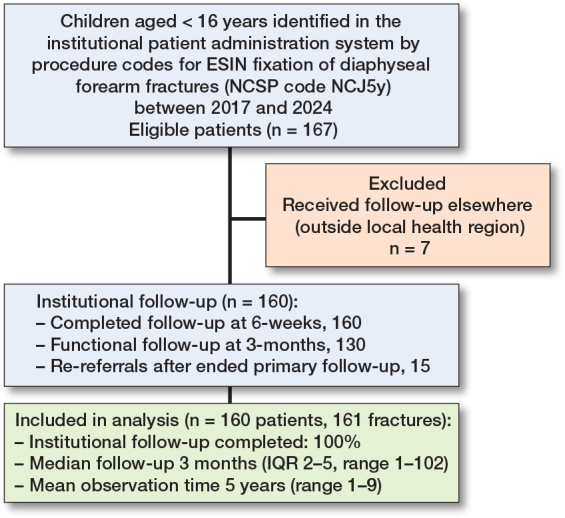
Flowchart of patient selection and institutional follow-up.

Of 167 identified patients, 7 were excluded because follow-up took place outside the catchment area, leaving 160 children with 161 forearm fractures for inclusion. After completion of primary follow-up, 15 children (9.4%) were re-referred for local symptoms, resulting in a median clinical follow-up of 3 months (IQR 2–5; range 1.5–102) (Figure 2). The median age at index operation was 7.9 years, and 58% were boys. Closed reduction was performed in 87%, fixation of both bones in 76%, and a distal lateral approach was used for the radial nail in 97%. In total, 283 nails were implanted (mean diameter 2.2 mm). Baseline characteristics are presented in Table 1. Data was complete for all predefined variables.

**Table 1:** Baseline characteristics (n = 160 patients/161 fractures). Values are n (%) unless otherwise stated

Variable	Value
Demographics	
Age, mean (range) years	7.9 (2–15)
Sex	
Boys	93 (58)
Girls	67 (42)
Side	
Left	96 (60)
Right	63 (39)
Bilateral	1 (0.6)
Fracture characteristics	
Fracture location	
Both bones	122 (76)
Radius	25 (16)
Ulna	14 (8.7)
Open fractures	0 (0)
Treatment details	
Reduction method	
Closed	140 (87)
Open	21 (13)
Number of nails	
1 nail	39 (24)
2 nails	122 (76)
Nail diameter, mean (range) mm	2.2 (1.5–3.0)
Nails implanted	
Total	283 (100)
Radius	147 (52)
Ulna	136 (48)
Distal radial approach	
Lateral	143 (97)
Dorsal	4 (2.7)
Operative time (index procedure), mean (CI), minutes	56 (52–60)

CI = 95% confidence interval.

### Implant retention and nail protrusion

At a mean observation time of 5 years (range 1–9), 132/160 children (83%, CI 76–88) retained their implants (Table 2). 241/283 nails (85%, CI 81–89) were retained, with no difference in retention between ulnar and radial nails (P = 0.3).

**Table 2:** Nail protrusion and retention with comparisons

Variable	n	Median value (IQR)	P value	Retention rate		OR (CI)	P value
				n/N	% (CI)		
All patients	160	3.3 (0.0–7.2) mm		132/160	83 (76–88)	NA	NA
All nails	283	3.3 (0.0–7.2) mm		241/283	85 (81–89)	NA	NA
By bone			0.1				
Radial nails	147	3.4 (0.0–8.1) mm		122/147	83 (76–88)	0.70 (0.36–1.36)	0.3
Ulnar nails	136	3.0 (0.0–6.4) mm		119/136	87 (76–89)		
By outcome			< 0.001				
Retained nails	241	2.6 (0.0–6.3) mm		NA		NA	NA
Removed nails due to irritation	20	7.6 (4.7–11.4) mm		NA		NA	NA
By timing			0.09				
Early removed nails due to irritation	10	10.7 (4.6–15.5) mm		NA		NA	NA
Later removed nails due to irritation	10	5.4 (4.4–8.0) mm		NA		NA	NA
By operative time			0.9				
Early removed nails due to irritation	10	37 (24–54) min		NA		NA	NA
Later removed nails due to irritation	10	34 (24–55) min		NA		NA	NA
By protrusion							
No protrusion	115	NA	NA	NA		NA	NA
Protrusion < 3.3 mm **^a^**	133	NA	NA	133/133	100 (97–100)	NA	NA
Protrusion > 3.3 mm **^a^**	128	NA	NA	108/128	84 (77–89)	NA	NA

CI = 95% confidence interval; IQR = interquartile range; OR = odds ratio.

“Early” refers to within 1 year after the index operation.

a Nails removed for reasons other than irritation are excluded

Median nail protrusion was 3.3 mm (IQR 0.0–7.2), with no significant difference in nail protrusion between the forearm bones (P = 0.1). Retained nails had significantly lower protrusion than nails removed for irritation (2.6 mm vs 7.6 mm; P < 0.001). Among nails removed for irritation, protrusion was numerically lower in late than early removals (5.4 mm vs 10.7 mm), although this difference was not statistically significant (P = 0.09). Operative time did not differ between these early and late removals (P = 0.9).

**Figure 3:**
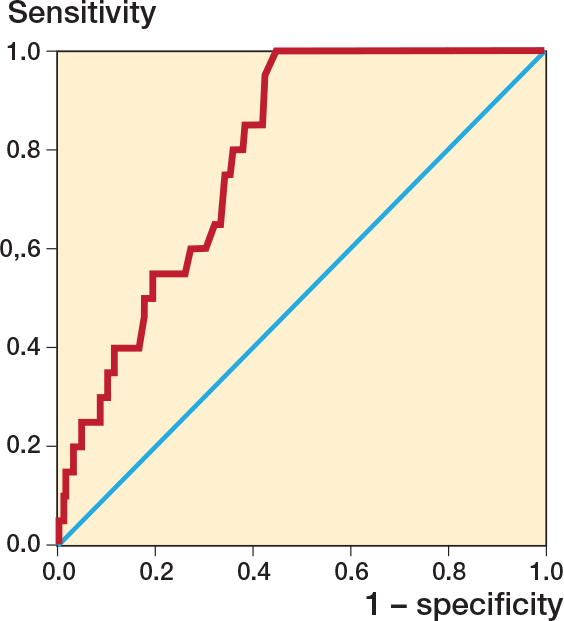
Receiver-operating-characteristic (ROC) curve for nail protrusion predicting local irritation requiring implant removal, with the diagonal reference line indicating no discrimination. The area under the curve (AUC) was 0.79 (P < 0.001), with an optimal cut-off of 3.3 mm (Youden’s index 0.55; sensitivity 100%, 95% confidence interval [CI] 83–100, specificity 55%, CI 49–61).

ROC analysis identified a protrusion threshold of 3.3 mm for predicting irritation-related implant removal (AUC 0.79, CI 0.71–0.86). No irritation-related removals occurred below this threshold, corresponding to a sensitivity of 100% (CI 83–100). At this ROC-derived threshold, specificity was 55% (CI 49–61). The Youden index was 0.55 (Figure 3). At this threshold, the positive predictive value was 16% and the negative predictive value was 100%. Nail diameter showed no discriminative ability to predict irritation-related removal (AUC 0.48, P = 0.7).

### Secondary procedures and complications

**Figure 4:**
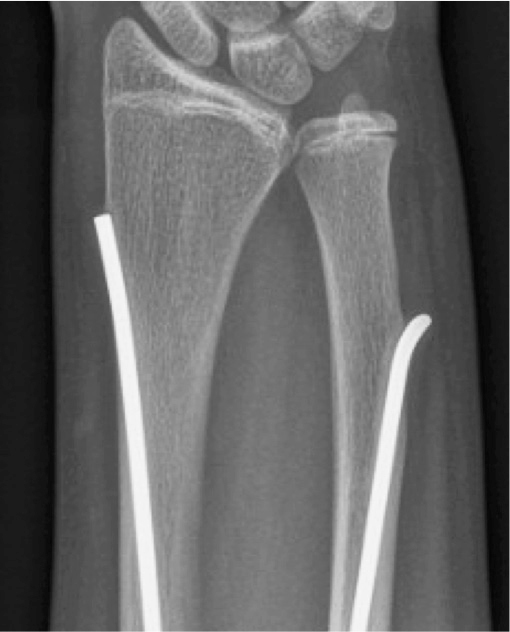
Anteroposterior radiographs illustrating the single failed attempt at implant removal. Removal was attempted 37 months after the index surgery due to local complaints from an ulnar nail with distal perforation during growth. Despite distal ulnar grip, extraction was not possible because the nail was firmly seated. The nail tip was cut instead, and symptoms resolved despite the nail not being removed.

Secondary procedures and complications occurred in 48/160 patients (30%, CI 23–37), and unplanned secondary procedures were performed in 30/160 (19%, CI 13–26), mostly involving implant removal (Table 3). Local irritation was the most common reason for secondary procedures (16/160; 10%, CI 6–16), occurring both early and late. Refracture occurred in 9/160 patients (6%, CI 3–10). Most occurred early, with a single late refracture after 36 months affecting the radius and involving the ulna at the distal nail end. Treatment consisted of implant removal and revision fixation, as in most refractures. All attempted implant removals were successful except in 1 patient in whom distal ulnar nail shortening was performed due to cortical perforation during growth (Figure 4).

**Table 3:** Distribution of complications with treatment and clinical course

Event	Patients		Treatment ^a^	Clinical course at last follow-up (with explanation)
Onset (months)	n	% (CI)		
All events	48	30 (23–37)	Unplanned secondary procedure/Conservative management	Resolved or improving
Secondary procedures	30	19 (13–26)	Unplanned secondary procedure	Resolved or improving Resolved
Local irritation	16	10 (6–16)		
Early: (4–8)	7		TEN removal (radial): 14 **^b^**	
Late: (15–98)	9		TEN removal (ulnar): 5 TEN shortening (ulnar): 1	
Refracture	9	6 (3–10)		Resolved or improving
Early: (1–7)	8 1		Closed reduction only: 1 TEN removal: 1 Revision TEN: 3 Plate fixation: 3	Resolved Improving (remodeling at 17 months) Resolved Resolved
Late: (36)	1		Revision TEN	Resolved
Infection (early)	3	2		Resolved
Radial wound infections	2		TEN removal	
Ulnar osteomyelitis	1		TEN removal + debridement **^c^**	
Malalignment (early)	1	1	TEN Removal	Resolved
Nerve deficit (early)	1	1	Nerve exploration	Resolved
Posterior interosseous nerve deficit				
Complications	19	12 (7–18)	Conservative management	Normalized or improving
Nerve deficits (early)	11	7		
Sensory: radial	7	4		Normalized or improving (at 1–3 months)
After TEN implantation	6			Improving (at 1–3 months)
After TEN removal **^b^**	1			
Sensorimotor: 2 radial,				
1 ulnar, 1 median	4	3		Normalized or improving at 3–4 months
After TEN implantation	4			
Late local irritation: (12–56)	6	4		No indication for intervention (4 without protrusion)
Reduced forearm rotations (early)	2	1		Resolved

TEN = titanium elastic nail.

a Unplanned implant removal for local irritation was symptom-driven and could involve removal of 1 or both nails. In cases of refracture, infection, or malalignment, unplanned implant removal was complication-driven and involved removal of all nails (1 or 2). In refractures, revision procedures included replacement with new TEN(s). In cases of infection or malalignment, TEN removal was performed to prevent progression of infection or to facilitate remodeling, respectively.

b A single patient experienced 2 complications: local irritation and a sensory radial nerve deficit after TEN removal.

c A single patient experienced 2 secondary procedures with debridement after TEN removal.

Complications occurred in 19/160 patients (12%, CI 7–18). Postoperative nerve symptoms were most common (11/160; 6.9%), primarily involving the superficial radial nerve (7/160; 4.4%) after implant insertion or removal. Late-onset local discomfort without subsequent implant removal occurred in 6/160 patients (3.8%), most without measurable protrusion.

Other events (infection, malalignment, and reduced range of forearm rotation) were uncommon and occurred early. All sequelae from secondary procedures and complications had resolved or were improving at last clinical follow-up.

## Discussion

We aimed to evaluate whether ESIN impaction permits implant retention in pediatric forearm fractures. We found that implant retention was high, at both the patient and implant level (83–85%), and a low radiographic protrusion threshold (3.3 mm) was associated with irritation-related implant removal. Irritation requiring removal occurred in a minority of patients (10%), and late symptoms were uncommon with retained implants (3.8%). Other events consisted mainly of early refractures and superficial radial nerve symptoms. Our findings extend existing knowledge by demonstrating high implant retention in a large forearm cohort with long-term observation after impaction, an under-studied aspect of ESIN management.

Previous evidence on implant retention after ESIN remains limited. Morshed et al. reported a 75% retention rate after femoral ESIN, with residual discomfort in 25% after a mean follow-up of 3.6 years [12]. In the present larger forearm cohort, retention was higher at both the patient and implant level (83–85%), and persistent symptoms without removal were uncommon after longer observation.

Protrusion is a recognized cause of soft-tissue irritation [5,15], necessitating implant removal [11,12]. Narayanan et al. reported increased removal above a 10-mm cut-off in a relatively small femoral cohort (n = 77) with a median follow-up of 10 months [11]. In our study, irritation-related removals occurred at a low median protrusion value (7.6 mm). The 3.3 mm threshold distinguished retained implants from those removed for irritation in this cohort with longer observation time.

Reported complication rates after ESIN range from 9% to 69% [1]. Local irritation has been reported in up to 19% before routine removal at 6 months [5]. Refractures occur in approximately 5%, mostly before or shortly after removal, but occasionally later (within 2 years after implant removal) [10]. Postoperative nerve symptoms have been noted in 12%, with superficial radial nerve involvement in 3% [13]. Complications related to implant removal, including failed extraction, have been reported in 4% around 6 months [9]. Both retention time and protrusion length may be associated with difficulties in ESIN removal [16]. Other ESIN-related complications are uncommon [3]. In the present cohort, the overall secondary procedure and complication rate (30%) fell within this reported range, and the frequency of individual events was consistent with previous estimates.

The principal finding is that long-term implant retention after impacted forearm ESIN is achievable and closely related to nail protrusion. Local irritation occurred at expected frequency but presented as irritation-related implant removal in the long term rather than before routine implant removal, likely reflecting reduced nail protrusion after impaction. The high retention rate is unlikely to reflect persistent or unrecognized symptoms, as few patients were re-referred for local complaints and late implant removals were rare. Our findings suggest that nails protruding below 3.3 mm carry a low risk of irritation, whereas those above may become symptomatic. This corresponds approximately to the nail diameter (2.2 mm), providing a simple intraoperative reference for limiting protrusion. The threshold should therefore be interpreted as a safety margin rather than a precise predictor. Its precision may be influenced by follow-up duration, sample size, and possibly fracture location, similar to the retention estimate. Protrusion was not lower in late than in early removals, suggesting that other factors, such as differences in soft-tissue coverage, may influence when irritation from friction between the nail end and surrounding soft tissue develops.

Other events may reflect different mechanisms. The early occurrence of refractures is consistent with mechanical overload at the fracture site during new trauma before full consolidation. The solitary late refracture showed a nail-related pattern, with possible overload at the nail tip after consolidation. The low rate of superficial radial nerve symptoms likely reflects adequate exposure during nail insertion and removal, whereas other nerve symptoms may be related to closed-reduction maneuvers, as previously suggested [13]. Nails extracted for irritation had protrusion above the threshold, providing a sufficient grip, which may explain the ease of these extractions.

Apart from irritation-related implant removal in the current study (10%), compared with routine removal in standard ESIN, the overall event burden was comparable. Refractures occurred mainly early and at the expected frequency, suggesting no apparent effect of prolonged retention. Likewise, postoperative nerve symptoms and other uncommon complications (infection, malalignment, and reduced range of forearm rotation) occurred without any apparent link to impaction. Although 1 extraction failed after prolonged retention, the duration of late removal procedures was not increased, indicating that delayed removal is generally feasible when required. Overall, the observed events appear to be related to standard ESIN rather than to impaction, prolonged retention, or delayed extraction, supporting the safety of this technique.

Further interpretation concerns the applicability of the impaction technique. Nail diameter did not predict irritation-related implant removal, suggesting that impaction is applicable across implant sizes and forearms of different ages. In contrast, lateral impaction of a short nail end against the cortex may become technically more demanding with increasing nail diameter.

### Strengths

Strengths include the relatively large, consecutive, population-based cohort with long-term observation and treatment by on-call residents.

### Limitations

Selection bias during primary follow-up may have happened but is suggested to be low due to consecutive inclusion at a single tertiary center serving a defined catchment area. Later follow-up relied on re-referral, which may have underestimated minor symptoms among patients without implant removal. Information bias is likely limited by standardized data extraction from complete medical records. Radiographic measurements followed published methods [5,11,15], although the absence of formal reliability testing may have introduced imprecision. Confounding is likely limited, as nail protrusion reflects surgical technique. Multivariable adjustment was not feasible due to few removals, and ROC analysis was therefore used to evaluate discrimination. Besides the absence of reliability testing of radiographic measurements and passive re-referral for long-term follow-up, additional limitations include the lack of a control group and the limited sample size with only unadjusted subgroup comparisons. In addition, the analysis focused on mechanical aspects of nail protrusion as a cause of irritation, while other mechanisms, such as allergic reactions, were not specifically assessed. These limitations may have underestimated very late or minor complications, including technical difficulties at delayed implant removal, and may have introduced radiographic measurement imprecision, particularly given the low threshold. They also preclude conclusions regarding superiority or equivalence, as any comparison with standard ESIN is indirect. The heterogeneous fracture cohort supports transferability to forearm shaft fractures. The exploratory ROC-derived threshold is, however, cohort- and follow-up-dependent and should therefore be interpreted with caution when generalizing to other anatomical segments, bones, or implant systems.

### Conclusion

Impaction to reduce nail protrusion was associated with high long-term implant retention, without complications beyond those expected with standard ESIN. A protrusion threshold of approximately 3 mm was associated with irritation-related implant removal and may support clinical decision-making regarding further ESIN impaction to achieve retention. These findings support impacted ESIN as a safe and practical refinement in the treatment of pediatric diaphyseal forearm fractures.

## Notes

VH and JEB planned and designed the study. JEB contributed to data verification, performed the analyses and drafted the manuscript. CA contributed to data extraction, manuscript writing and revision. CF assisted in data extraction. AR contributed to data extraction and performed radiographic measurements. All authors approved the final version.

Handling co-editors: Ilkka Helenius and Robin Christensen

*Acta* thanks Søren Kold, Klaus Dieter Parsch and Antti Joonas Stenroos for help with peer review of this manuscript.

## References

[1] Reddy E, Sriwastwa A, Patel S, Gupta R, Parikh S N. Elastic stable intramedullary nailing for pediatric forearm fractures: a review article. J Clin Orthop Trauma 2025; 71: 103249. doi: 10.1016/j.jcot.2025.103249. 41245351 PMC12613074

[2] Hunt A, Judkins N, Biggs A, Sedgwick P, Hing C B, Yeo A. The use of flexible nails in the treatment of paediatric long bone fractures: experience at a level one paediatric trauma centre, a cohort study. J Clin Orthop Trauma 2024; 49:102355. doi: 10.1016/j.jcot.2024.102355. 38356689 PMC10863312

[3] Poutoglidou F, Metaxiotis D, Kazas C, Alvanos D, Mpeletsiotis A. Complications of elastic stable intramedullary nailing in pediatric forearm fractures, a systematic review. J Orthop 2020; 20: 125-130. doi: 10.1016/j.jor.2020.01.002. 32025135 PMC6997658

[4] Lascombes P, Haumont T, Journeau P. Use and abuse of flexible intramedullary nailing in children and adolescents. J Pediatr Orthop 2006; 26(6): 827-34. doi: 10.1097/01.bpo.0000235397.64783.d6. 17065959

[5] Nisar A, Bhosale A, Madan S S, Flowers M J, Fernandes J A, Jones S. Complications of Elastic Stable Intramedullary Nailing for treating paediatric long bone fractures. J Orthop 2013; 10(1): 17-24. doi: 10.1016/j.jor.2013.01.003. 24403743 PMC3768244

[6] Ligier J N, Metaizeau J P, Prévot J, Lascombes P. Elastic stable intramedullary nailing of femoral shaft fractures in children. J Bone Joint Surg Br 1988; 70(1): 74-7. doi: 10.1302/0301-620X.70B1.3339064. 3339064

[7] Peterson H A. Metallic implant removal in children. J Pediatr Orthop 2005; 25(1): 107-15. doi: 10.1097/00004694-200501000-00024. 15614071

[8] Busam M L, Esther R J, Obremskey W T. Hardware removal: indications and expectations. J Am Acad Orthop Surg 2006; 14(2): 113-20. doi: 10.5435/00124635-200602000-00006. 16467186

[9] Simanovsky N, Tair M A, Simanovsky N, Porat S. Removal of flexible titanium nails in children. J Pediatr Orthop 2006; 26(2): 188-92. doi: 10.1097/01.bpo.0000218534.51609.aa. 16557132

[10] Fernandez F F, Langendörfer M, Wirth T, Eberhardt O. Failures and complications in intramedullary nailing of children’s forearm fractures. J Child Orthop 2010; 4(2): 159-67. doi: 10.1007/s11832-010-0245-y. 21455473 PMC2839862

[11] Narayanan U G, Hyman J E, Wainwright A M, Rang M, Alman B A. Complications of elastic stable intramedullary nail fixation of pediatric femoral fractures, and how to avoid them. J Pediatr Orthop 2004; 24(4): 363-9. doi: 10.1097/00004694-200407000-00004. 15205616

[12] Morshed S, Humphrey M, Corrales L A, Millett M, Hoffinger S A. Retention of flexible intramedullary nails following treatment of pediatric femur fractures. Arch Orthop Trauma Surg 2007; 127(7): 509-14. doi: 10.1007/s00402-007-0286-y. 17237933

[13] Lyman A, Wenger D, Landin L. Pediatric diaphyseal forearm fractures: epidemiology and treatment in an urban population during a 10-year period, with special attention to titanium elastic nailing and its complications. J Pediatr Orthop B 2016; 25(5): 439-46. doi: 10.1097/BPB.0000000000000278. 26919620

[14] von Elm E, Altman D G, Egger M, Pocock S J, Gøtzsche P C, Vandenbroucke J P; STROBE Initiative. The Strengthening the Reporting of Observational Studies in Epidemiology (STROBE) statement: guidelines for reporting observational studies. Lancet 2007; 370(9596): 1453-7. doi:10.1016/S0140-6736(07)61602-X. 18064739

[15] Luhmann S J, Schootman M, Schoenecker P L, Dobbs M B, Gordon J E. Complications of titanium elastic nails for pediatric femoral shaft fractures. J Pediatr Orthop 2003; 23(4): 443-7. PMID: 12826940. 12826940

[16] Liu J, Su Y. Factors which can influence elastic stable intramedullary nailing removal in healed bone cysts in children. Sci Rep 2024; 14(1): 11129. doi: 10.1038/s41598-024-61828-3. 38750240 PMC11096159

